# A Window into the Brain: Advances in Psychiatric fMRI

**DOI:** 10.1155/2015/542467

**Published:** 2015-08-27

**Authors:** Xiaoyan Zhan, Rongjun Yu

**Affiliations:** ^1^School of Psychology and Center for Studies of Psychological Application, South China Normal University, 55 West Zhongshan Avenue, Tianhe District, Guangzhou 510631, China; ^2^School of Economics & Management and Scientific Laboratory of Economic Behaviors, South China Normal University, 55 West Zhongshan Avenue, Tianhe District, Guangzhou 510631, China

## Abstract

Functional magnetic resonance imaging (fMRI) plays a key role in modern psychiatric research. It provides a means to assay differences in brain systems that underlie psychiatric illness, treatment response, and properties of brain structure and function that convey risk factor for mental diseases. Here we review recent advances in fMRI methods in general use and progress made in understanding the neural basis of mental illness. Drawing on concepts and findings from psychiatric fMRI, we propose that mental illness may not be associated with abnormalities in specific local regions but rather corresponds to variation in the overall organization of functional communication throughout the brain network. Future research may need to integrate neuroimaging information drawn from different analysis methods and delineate spatial and temporal patterns of brain responses that are specific to certain types of psychiatric disorders.

## 1. Introduction

The human brain is the most mysterious and vital organ. Recent neuroimaging techniques, including functional magnetic resonance imaging (fMRI), electroencephalography (EEG), and magnetoencephalography (MEG), now allow us to probe the brain at unprecedentedly high temporal or spatial resolution without the use of invasive techniques. Since the first fMRI brain scans of the 1980s, scientists have achieved great progress not only in technical procedures employed to acquire brain imaging data but also in data processing methods which subsequently reveal an inspiring understanding of the brain drawn from various data perspectives. fMRI has become the dominant technique in neuroimaging due to its noninvasiveness, lack of radiation exposure, a relatively good spatial and temporal resolution, and relative ease to acquire.

**Figure 1:**
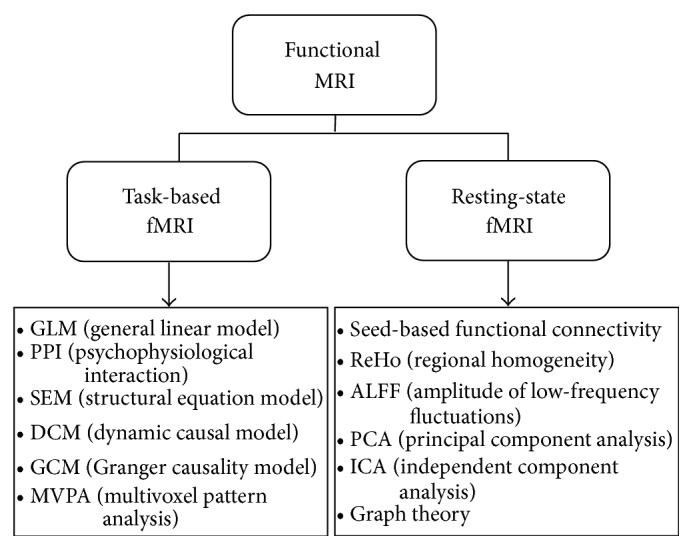
A summary of mainstream fMRI neuroimaging methods.

In this paper, we will review popular data processing methods used in task-based fMRI and resting-state fMRI (see Figure 1 for a summary of mainstream fMRI methods). Some methods introduced in task-based fMRI, such as MVPA, are also applied in the case of resting-state fMRI data. Different analysis methods probe specific brain activity patterns. The application of these methods to investigating psychiatric disorders will be discussed in great detail. We also point out here that within neuropsychology there is an ongoing paradigm shift from identifying foci of abnormalities to delineating the functional connectivity among several brain regions, towards developing a global understanding of aberrations at the level of large-scale, whole brain networks. The advantages and disadvantages of each neuroimaging method are discussed and compared in order to help researchers select the methods most appropriate to their purposes.

## 2. Task-Based fMRI

Due to the great sensitivity of fMRI signals to event-related changes in neuronal blood flow, we can compare the BOLD signal differences between patients with psychiatric disorders and normal subjects when performing different kinds of tasks in order to elucidate how a brain in a state of disorder functions differently from a normal one. In this section, we will introduce several methods frequently used in task-related fMRI analyses and discuss the advantages and disadvantages of each method (see Table 1). Also note that these techniques are not mutually exclusive, such that two or more of them may be applied to the same dataset according to the quality of the dataset and the purpose of the study. Of importance, we discussed how these analyses can inform each other and some caveats in using these methods, such as choosing the frequency bands in resting-state fMRI (R-fMRI).

### 2.1. Subtraction and Correlation

**Figure 2:**
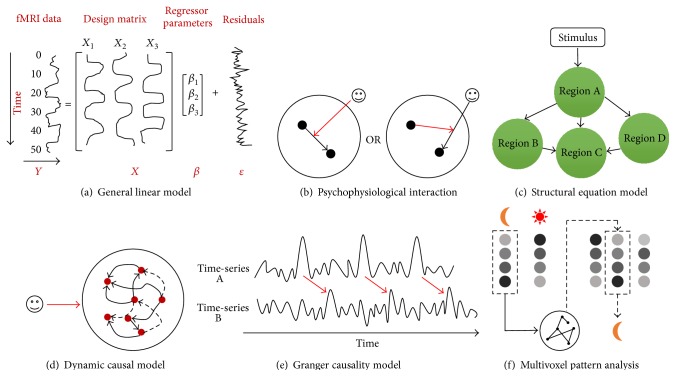
A summary of task-related fMRI analysis methods. (a) An example of the general linear model containing the BOLD signal time-series *Y* within a particular voxel, the design matrix *X* including three regressors of interest, the regressor parameters *β*, and the unexplained residuals *ε*. (b) Psychophysiological interaction can identify how the connectivity between two particular brain regions is modulated by an experimental variable or how a specified region modulates the relationship between an experimental variable and another brain region. (c) Structural equation model includes the stimulus to have an influence on all variables without input within the model. (d) An illustration of the dynamic causal model. (e) An illustration of the Granger causality model. Time-series A can be said to cause time-series B because the pattern of B is similar to A and A has temporal precedence. (f) An example of multivoxel pattern analysis. A specific classifier is chosen to identify the pattern of a certain mental state, whereafter the accuracy of the classifier will be tested using a new dataset.

The general linear model (GLM) has gained growing popularity in task-related fMRI analysis since its introduction into the neuroimaging community by Friston et al. [1] in 1994, due to its easy interpretability and fast computability. GLM provides a framework for most kinds of data modeling and can minimize confounding factors, such as head motions or respiration from the subject, provided that these data are modeled. The aim of the general linear model is to explain the variation of the time course, *y*_1_,…, *y*_*i*_,…, *y*_*n*_, in terms of a linear combination of explanatory variables plus a Gaussian error term. The general linear model in matrix form can be written as

(1) $$\begin{array}{c} Y=X\beta +\varepsilon , \end{array}$$

where *Y* is the vector of observed pixel values, *β* is the vector of parameters, and *ε* is the vector of error terms. The matrix *X* is known as the design matrix (Figure 2(a)). It has one row for every time point in the original data and one column for every explanatory variable in the model. In the GLM, the columns of *X* contain vectors corresponding to the “on” and “off” elements of the stimulus presented. By finding the magnitude of the parameter in *β* corresponding to these vectors, the presence or absence of activation can be detected. The aim of GLM analysis is to identify the brain regions that show significant signal change in response to the experimental conditions. Each pixel is assigned a value dependent on the likelihood of the null hypothesis being false. The null hypothesis is that the observed signal changes can be explained purely by random variation in the data. The brain image containing such information for all voxels is called a statistical parametric map [1]. One of the simplest methods for obtaining results from an fMRI experiment is to perform a simple subtraction on two experimental conditions. By averaging together all the images acquired during the “on” phase of the task and subtracting the average of all the “off” images, brain regions that are activated during the “on” phase of the task can be drawn out of the data pool and identified. Using parametric design, researchers can also examine parametric correlation with behavior in the brain. GLM is also the base of the majority of functional/effective connectivity estimation techniques which will be introduced in the following sections.

GLM is the dominant method used in task-based fMRI. Studies on psychiatric disorders have used this method to compare brain activities induced by certain experimental manipulations in the patient group and in the control group. For example, Juckel et al. [2] scanned patients with schizophrenia and healthy subjects using fMRI while they performed a “monetary incentive delay” task, in which they anticipated potential monetary gain, loss, or neutral outcomes. Following preprocessing, the fMRI data was modeled by GLM with three explanatory variables (“gain,” “loss,” and “neutral outcome,” indicating experimental cues) convolved with Cohen's gamma-function. Activations of different experimental conditions can be compared based on the BOLD response differences which can be assessed using linear combinations of the estimated GLM parameters (*β* values). Within-group activation (e.g., “gain versus neutral outcome”) and intergroup differences can be compared by including the BOLD response variations of all subjects in each group in a second-level random effects analysis. However, GLM has also undergone some criticism focusing primarily on the assumptions the model makes [3]. Greater attention should be paid to checking the model's assumptions when applying GLM as a tool to analyze task-related fMRI data. This approach is the main method used in task-based fMRI in psychiatric research.

### 2.2. Psychophysiological Interaction (PPI)

One important goal of neuroimaging research is to describe the pattern of brain connectivity among different regions. Functional connectivity refers to undirected associations between brain regions while effective connectivity reveals a directed and causal relationship. Psychophysiological interaction, in a clever use of the GLM, measures how functional connectivity is affected by psychological variables without specifying the directions of such influences [4]. It examines how brain activity can be explained by the interaction between an experimental variable (e.g., level of attention) and the coupling between signals from a particular brain area (the source area) and signals from voxels in the rest of the entire brain (Figure 2(b)). A psychophysiological interaction means that the contribution of one area to another changes significantly with the experimental or psychological context. In other words, regional responses in the source area to an experimental or psychological factor are modulated by signals from a distal brain region. Das et al. [5] used a behavioral task in which schizophrenia patients and healthy participants were asked to identify the emotions displayed on a series of facial images presented either supraliminally or subliminally during scanning. Subtraction analyses of fMRI data showed that, compared to healthy controls, schizophrenia patients showed reduced activity in the right amygdala and MPFC during conscious perception of fear (relative to neutral) and also in the bilateral amygdala and rostral ACC of the MPFC during subliminal perception of fear. PPI analyses revealed reduced neural activity in schizophrenia patients, relative to control subjects, in the pathway from the amygdala and its projection to the medial prefrontal cortex (MPFC) in response to fear perception. In another fMRI study, Wang et al. [6] applied PPI analysis to explore how abnormal functional connectivity in mPFC in schizophrenics altered as a result of psychological context or variables. They found that schizophrenic patients showed higher mPFC-LSTG connectivity under self-generated conditions than under other-generated conditions.

### 2.3. Structural Equation Model (SEM)

The structural equation model, which was developed in the field of econometrics and first applied to neuroimaging data in 1991 [7], is another way to measure effective connectivity. Like PPI, SEM is also used to describe how effective connectivity is affected by experimental context. But, compared to PPI, SEM is better at identifying causal relationships and it combines covariances in activity between different brain areas with anatomical models of these brain areas' connections [8, 9]. SEM contains a group of regions and a group of directed connections and these connections are presumed to represent causal relationships (Figure 2(c)). SEM requires an a priori assumption of causality without inference from the data and from this basis subsequently builds a model about how the regions are connected to each other. Free parameters in these models are “path coefficients”—representing the strength of connections. This approach offers a move from correlational analysis (inherently bidirectional) to unidirectional connections (paths) which imply causality. One well-known strength of SEM is the method's ability to specify latent variable models that provide separate estimates of relations among latent constructs and their manifest indicators (the measurement model) and of the relations among constructs (the structural model) [10]. Another strength of SEM is the availability of measures of global fit that can provide a summary evaluation of even complex models that involve a large number of linear equations [10]. It has proved useful in distinguishing a patient's neural network from a normal subject's neural network in one fMRI simulation study [11]. In another fMRI study, schizophrenic patients were scanned while performing a “2-back” working memory task. SEM was used to assess effective connectivity within a cortical-subcortical-cerebellar network for mnemonic information processing and comparison of group differences [12].

### 2.4. Dynamic Causal Model (DCM)

Similar to SEM, the dynamic causal model is also an approach to estimate effective connectivity and how this connectivity is influenced by experimental variables. However, underlying SEM and DCM are two very distinct generative models (see [10] for a comprehensive comparison of DCM and SEM). DCM treats the brain as a deterministic, dynamic system with a nonlinear and dynamic nature in which the observed BOLD signal recorded by fMRI results from changes in neuronal activity caused by external inputs [6, 13, 14], while SEM does not distinguish “neuronal” levels from “hemodynamic” levels and changes in effective connection lead directly to changes in the covariance structure of the observed hemodynamics in this method. Considering changes in effective connectivity in the brain occur at a neuronal level, DCM is a better method for fMRI analysis.

The goal of DCM is to estimate and make inferences about the coupling among brain areas and how that coupling is influenced by changes in experimental context by building a reasonably realistic neuronal model of interacting brain regions. This model is then supplemented with a forward model of how neuronal or synaptic activity is transformed into a measured response such as the BOLD signal [13]. This enables estimation of the parameters of effective connectivity from observed data. With DCM, a causal model is built in which neuronal activity in a certain region causes changes in neuronal activity in other regions through interregional connections and self-connections that can be modulated by experimental variables (Figure 2(d)). Effective connectivity is parameterized in terms of coupling among unobserved brain states (e.g., neuronal activity in different regions). The objective is to estimate these parameters by perturbing the system and measuring the response. In brief, the core of DCM distinguished from conventional approaches such as SEM and GCM is that it attempts to model neuronal interactions instead of signals [15] and explore the estimation problem according to the designed perturbations that accommodate experimental inputs. DCM has been broadly used in psychiatric fMRI. For example, in a stoke patient's fMRI study, Grefkes et al. [14] applied DCM of a bilateral network comprising M1, the lateral premotor cortex, and the supplementary motor area (SMA) to assess changes in the endogenous and task-dependent effective connectivity between the cortical motor areas activated by a hand movement task at baseline, following vertex stimulation and contralesional M1 stimulation with repetitive transcranial magnetic stimulation (rTMS). In another fMRI study, Roebroeck et al. [16] used DCM to examine the effects of Parkinson's disease and dopaminergic therapy and concluded that the DCM model selection is robust and sensitive enough to study clinical populations and their pharmacological treatment.

### 2.5. Granger Causality Model (GCM)

GCM is another popular method of estimating effective connectivity [16–18], based on the reasoning that one time-series can be considered to cause another if using the past information of the former can help forecast the latter better than only using the past information of the latter [19]. GCM can provide an estimate of connection directionality when one time-series resembles a time-shifted version of the other, supposing that one with temporal precedence caused the other [20] (Figure 2(e)). This method does not depend upon an a priori assumption of a structural model that contains preselected ROIs and connections between them, which differs from SEM with the goal of contrasting the predefined causal model with real datasets. Furthermore, GCM defines the causal relationship between two stochastic time-series relying purely on temporal precedence in their interdependency. Demirci et al. [21] scanned schizophrenic patients and healthy subjects with fMRI while performing a Sternberg item recognition paradigm (SIRP) and auditory oddball (AOD) tasks. The fMRI data were then decomposed into maximally independent spatial components and corresponding time courses by applying ICA. The time courses for each of the components that were most related to the cognitive task with the most important and meaningful activation patterns were then used as inputs to a Granger causality test that investigated group differences in causal relationships between independent components over a frequency spectrum. Granger causality can also be applied to resting-state fMRI data to infer instantaneous correlation and causal influences. Hamilton et al. [22] measured BOLD signals of patients suffering from major depressive disorder during resting state and found that hippocampal and vACC activation in depressed participants predicted subsequent decreases in dorsal cortical activity by applying GCM.

### 2.6. Multivoxel Pattern Analysis (MVPA)

MVPA is gaining increasing interest in the neuroimaging community because it allows us to detect differences between conditions with higher sensitivity than conventional univariate analysis by focusing on the analysis and comparison of distributed patterns of activity (Figure 2(f)). In such a multivariate approach, data from individual voxels within a region are jointly analyzed. MVPA applies pattern-classification algorithms like support vector machines (SVM) [23–27], neural networks [28–30], or linear discriminant analysis (LDA) [31, 32] as classifiers to distinguish spatial patterns of different mental states and decode the perceptual or cognitive states of an individual. In the analysis of fMRI data, the features that are descriptive of the objects are first chosen, whereafter a subset of these features to be used for classification is selected. The data is divided into two parts: a “training set” and a “testing set.” The pattern-classification algorithm utilizes the training set to train the classifier with the features and the prespecified classes of objects. The classifier thus “learns” a functional relationship between the features and the classes. Finally, the classification algorithm is tested for its generalization capabilities with the testing set. The percentage of correct classifications can be measured.

Like other multivariate approaches (e.g., PCA and ICA), MVPA takes into account multivoxel patterns of brain activity or connectivity. Information contained in these patterns can then be decoded by applying powerful pattern-classification algorithms. This method thus incorporates spatially distributed patterns of activity into the analysis, unlike univariate methods which treat every brain voxel independently. MVPA is often presented in the context of “brain reading” applications reporting that specific mental states or representational content can be decoded from fMRI activity patterns after performing a “training” or “learning phase.”

MVPA has been successfully applied to identify functional connectivity difference between males and females [23], patterns in perception of pain [33], moral intentions [34], consciousness [35, 36], and brain maturity [37]. In a study on subjects with autism spectrum disorder (ASD) conducted by Coutanche et al. [38], reliable correlations between MVPA classification performance and standardized measures of symptom severity that exceeded those observed using a univariate measure were found, which indicated MVPA had the potential to predict clinical symptom severity.

## 3. Resting-State fMRI Analyses

Brain regions which are active when our minds wander may hold a key to understanding neurological disorders and even consciousness itself. Resting-state fMRI, which measures spontaneous low-frequency fluctuations (<0.1 Hz) in the BOLD signal, is a relatively new pathway for evaluating regional interactions in the absence of tasks [39–41]. For a long time neuroscientists have thought that the brain enters a “quiet” state while a person is not doing anything but remaining still. However, the recent studies of resting-state fMRI reveal that there is a persistent level of background activity in the brain during rest, which is called “the default mode” (DM) [8, 11, 41–43]. Some neuroscientists believe that the default mode network (DMN) may be critical in uncovering the neural mechanism of psychiatric disorders ranging from Alzheimer's disease to depression [44–51]. On the other hand, due to its capacity for exploring individual differences, as well as its ease of acquisition, resting-state fMRI has become one of the most popular techniques in neuroimaging. In this section we will introduce several popular resting-state fMRI analysis methods and compare their advantages and disadvantages (see Table 2).

### 3.1. Seed-Based FC Analysis

**Figure 3:**
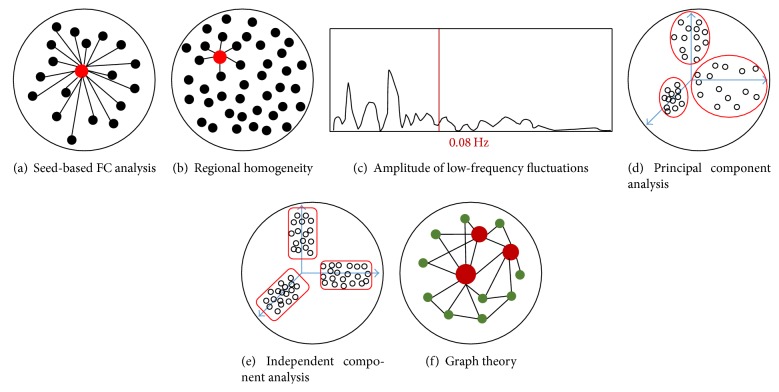
A summary of research analysis methods applied to resting-state functional MRI. (a) With seed-based functional connectivity analysis, a voxel or region is predefined and correlations are estimated between the selected “seed” and the remaining brain voxels. (b) An illustration of regional homogeneity (ReHo). (c) An illustration of amplitude of low-frequency fluctuations (ALFF). (d) Principal component analysis (PCA) transforms the original data into a new coordinate system where orthogonal variables are identified while retaining most of their variance. (e) Independent component analysis (ICA) is useful for searching a set of underlying sources of resting-state signals that are maximally independent of each other which can explain the resting-state patterns. (f) Graph theory views ROIs as nodes and correlations between them as the connectivity of the edges and then computes the connectional features of the graph.

The seed-based approach extracts BOLD time-series data from a “seed”—a priorly selected voxel or ROI—and assesses the correlation between the average BOLD signal of the seed and the time course of all other brain voxels (Figure 3(a)). Seed-based analysis has been applied in resting-state fMRI to explore the relationships between resting-state brain activity and motor response regions [39], intelligence [52], descent into sleep [53], cognitive decline in normal aging [54], memory [55], task-related activation correlated with schizophrenia [56], and task-positive and task-negative networks [57]. In an fMRI study conducted by Zhou et al. [58], to investigate patients with paranoid schizophrenia, the right dorsolateral prefrontal cortex and the posterior cingulate cortex were selected as two seed regions. Then, the investigators computed a correlation map by computing the correlation coefficients between the reference time-series in the seed region and the time-series from all other brain voxels from which they found abnormal interregional connectivity in the intrinsic organization in patients with paranoid schizophrenia. Parkinson's disease (PD) is characterized by motor symptoms resulting from the death of dopamine-generating cells. Previous studies on PD have been associated with abnormal task-related brain activation in sensory and motor regions as well as reward related network. In order to study corticostriatal skeletomotor circuit dysfunction in Parkinson's disease, in a recent resting-state fMRI study, the putamen and supplementary motor area (SMA) were selected as seed regions due to their roles in reward processing and motor control [59]. Enhanced putamen-SMA functional connectivity was also found in the PD group. Similarly, the periaqueductal gray (PAG) plays a key role in the descending modulation of pain and its functional connectivity has been intensively examined in chronic pain patients [60]. While seed-based FC analysis has the advantage of statistical transparency and comprehensible results, seed-based analysis also suffers from the potential biases attached to prior seed selection. For example, to examine the default network, researchers have used a variety of seeds and generated different versions of the default mode network [61].

### 3.2. Regional Homogeneity (ReHo)

ReHo is another straightforward technique that uses Kendall's coefficient concordance (KCC) to measure the similarity of a given voxel with its nearest neighbors based on the BOLD time-series [62] (Figure 3(b)). Multiple studies which applied ReHo to resting-state fMRI data processing have shown diminished ReHo of specific regions in heavy male smokers [63], patients with Alzheimer's disease [64], patients with depression [65, 66], patients with schizophrenia [67], patients with Parkinson's disease [68], children with ASD [69, 70], adults with ADHD [49], and normal aging people [68]. On the contrary, a positive correlation has also been found between ReHo of certain regions and intelligence [71], early blindness [72], and internet addiction disorder [73]. ReHo is very useful in identifying regional abnormality in psychiatric disorders, which may guide further network based analysis. For example, a recent study found that ReHo changes in schizophrenia are widespread [74], leading to brain-wide network analysis in schizophrenia [75, 76].

### 3.3. Amplitude of Low-Frequency Fluctuations (ALFF)

ALFF is an index that reflects the intensity of regional spontaneous brain activity by calculating the voxel-wise magnitude within a defined low-frequency range (Figure 3(c)). In order to reduce ALFF's sensitivity to physiological noise, Zou et al. [77] proposed a fractional ALFF (fALFF) approach calculating the ratio of power spectrum of low-frequency (0.01–0.08 Hz) to that of the entire frequency range. A number of resting-state fMRI studies have observed higher ALFF in the DMN areas than other areas [77–79]. Applications of ALFF in studies of conditions like depression [80], ADHD [81], PTSD [82], normal aging [83], and schizophrenia [84] have also revealed some exciting findings. Recently, by decomposing R-fMRI low-frequency (typically 0.01–0.1 Hz) oscillations (LFOs) into two distinct frequency bands [slow-5 (0.01–0.027 Hz), slow-4 (0.027–0.073 Hz)], researchers found that LFO amplitudes in the slow-4 band were higher than those in the slow-5 in many brain regions [85, 86]. Yu et al. (2014) further demonstrated that the abnormalities of LFOs in schizophrenia are dependent on the frequency band and suggest that future studies should take the different frequency bands into account when measuring intrinsic brain activity [85].

### 3.4. Principal Component Analysis (PCA)

PCA is a data-driven method that does not require the input of any prior information about the connectivity pattern. It has been found useful in estimating whether there are functional regions with correlated signal responses in human brain mapping [87]. It was first formulated by Pearson [88] and then developed as a useful technique for reducing the dimensionality of complex data sets and for extracting new orthogonal variables identified as principal components [89]. The basic idea of PCA is to find a set of orthogonal bases that can maximize the variance of data and to separate out the most meaningful information from the noise so as to uncover the hidden structure (Figure 3(d)). For fMRI data, PCA has the advantage of verifying the facticity of differences in the activations between conditions or groups without specifying any prior knowledge of the form of BOLD response or the structure of the experimental design [90]. It is often applied in psychiatric fMRI analysis combined with other techniques such as ICA and MVPA. For example, Shen et al. [91] aimed at classifying individuals into schizophrenic and healthy control groups by a quantitative method. They collected fMRI data from patients with schizophrenia and healthy subjects and reduced the data size by using PCA decomposition. Then ICA was employed to extract data on the functionally connected networks in the brain, yielding less noisy components, which would be used as input to the classifier algorithm. However, the effectiveness of PCA is based on strong assumptions like linearity, orthogonal principal components, and high signal noise ratio (SNR) [92]. Sometimes data sets cannot be said to fit within these assumptions.

### 3.5. Independent Component Analysis (ICA)

As an extension of PCA, ICA is likewise a data-driven method that has been successfully used in describing fMRI data [93–96]. With the identical goal of finding a new set of variables with lesser redundancy that would provide the best possible representation of observed phenomena, ICA measures redundancy by the much richer concept of independence (Figure 3(e)) and only requires relatively weak assumptions about the independence of source signals compared with PCA, which extracts interested variables based on decorrelation and requires some stringent assumptions [97]. The independent components are assumed statistically independent in ICA. One of the most useful applications of ICA is reducing the negative effects of artifacts for standard GLM-based analysis by using decomposition information [98, 99]. Another useful application of ICA is in detecting the resting-state functional connectivity and identifying RSNs (resting-state networks) [39, 94, 96, 100, 101]. Besides, ICA is also used in task-related fMRI group analysis called FENICA [102]. ICA has been widely applied to the study of brain diseases, such as Alzheimer's disease [20, 44], schizophrenia [21, 22], bipolar disorder [2, 103], and epilepsy [2].

### 3.6. Graph Theory

A hot recent method used in resting-state fMRI is graph theory. Graph theory is a mathematical theory and approach to studying graphs made up of nodes and edges and how these nodes connected by edges interact with each other [58, 104] (Figure 3(f)). The brain network can be described as being analogous to a graph in which voxels can be viewed as nodes and connections between voxels as edges [105]. In fMRI studies, graph theory has been used by some ambitious researchers seeking to present a comprehensive map of how the brain is organized. The unique characteristic of graph theory compared with the more traditional univariate fMRI methods is that graph theory can serve as a tool to directly describe and compare different brain networks utilizing topological parameters such as clustering-coefficient, characteristic path length, degree of connectivity, centrality, and modularity [106]. Evidence from graph theory in fMRI studies has shown that the brain is structured in a highly efficient organization with both a small-world topology achieved through the presence of hubs and a scale-free topology [107, 108]. Graph theory has been applied not only to resting-state fMRI and task-based fMRI so as to analyze the topology of functional brain networks [105, 109] but also to studies of cortical thickness [110, 111], surface area, and diffusion weighted imaging data [91, 112, 113] so as to analyze the topology of structural brain networks. These studies have illustrated an alteration of arrangements in structural and functional brain networks associated with normal aging [114, 115], multiple sclerosis [116, 117], Alzheimer's disease [118–120], schizophrenia [121–123], depression [124, 125], and epilepsy [110, 126].

## 4. Conclusion

Over the past decades, the development of fMRI techniques has made great contributions to our understanding of the neural mechanism underlying psychiatric disorder. In the present review, we summarize several major MRI methods widely used in psychiatric neuroimaging. Some methods such as ReHo and VBM focus on regional changes, whereas others take a systematic approach and emphasize the whole brain network. These methods together can reveal the abnormalities in brain structures and functions in psychiatric disorders. However, the functional significance of many measures such as ReHo and ALFF is still not well understood. Psychiatric disorders may be associated with very subtle changes in the brain. One single method may not be enough to fully capture the nature of such alternations. A systematic approach using multimodal neuroimaging and a variety of analysis methods has the potential to identify reliable biomarkers for specific psychiatric disorders. With ongoing progress being made in neuroimaging methods, neuroimaging holds clear promise in helping to diagnose and quantify psychiatric diseases.

## Figures and Tables

**Table 1:** Comparisons among different task-based fMRI analysis methods.

Methods	Purposes	Strengths	Limitations
General linear model	Estimating to what extent each known predictor contributes to the variability observed in the voxel's BOLD signal time course	(i) Mathematically simple, easily interpreted, and readily available in standard packages (e.g., the SPM software) (ii) Flexible to incorporate multiple quantitative and qualitative independent variables, such as low- frequency drifts and head motion	(i) Relies on assumptions such as appropriate repressors in the matrix and normality of the fMRI noise which are difficult to check

Psychophysiological interaction	“Searching” for regions that correlate differently with a particular region under certain experimental context	(i) Can explore the connectivity of the source area to the rest of the brain and how it interacts with the psychological variables	(i) Only involves one region of interest in one model (ii) Limited in the extent to which you can infer a causal relationship

Structural equation model	Estimating the degree to which the activity between different brain regions is connected and how this connectivity is affected by an experimental variable	(i) Can examine interactions of several regions of interest simultaneously and offer estimations of causal relationships (ii) Predetermined connections are based on prior anatomical or functional knowledge	(i) Causality is predetermined, and this might overlook several aspects of neural activity (ii) Assumes the interactions are linear (i.e., structural equation models are not time-series) (iii) Lacks temporal information

Dynamic causal model	Estimating and making inferences about the coupling among brain regions and how this coupling is affected by changes in experimental context at the neuronal level	(i) Biologically more accurate and realistic than other methods because DCM models interactions at the neuronal rather than the hemodynamic level and complex connectivity patterns between regions can be arbitrarily postulated	(i) Prespecified models are needed (ii) Requires much longer time to estimate parameters than SEM (iii) Neurodynamics in each region are characterized by a single state variable (“neuronal activity”)

Granger causality model	Measuring the predictability of one neural time-series from another	(i) No a priori specification of a model is needed. Thus this model can complement the hypothesis-driven methods and help to form directed graph models of regions and their interactions	(i) The causal relationship may be caused by the differences in hemodynamic latencies in different parts of the brain if long repetition times (TR) are used

Multivoxel pattern analysis	Applying pattern- classification algorithms to demonstrate the relationship between measures of brain activity and a perceptual state and provide an information-theoretic framework for the isolation of regions that uniquely represent a behavior	(i) Simultaneously examines the disparate signals carried within a set of voxels rather than examining individual voxels in parallel (ii) Can decode more complex information due to improved sensitivity and use of spatial information	(i) The possibility of overfitting increases as the classifier becomes more complex, which may result in poor performance in tests of generalization

**Table 2:** Comparisons among resting-state fMRI analysis methods.

Methods	Purposes	Strengths	Limitations
Seed-based FC analysis	Estimating correlations between the predefined voxel or regions and the rest of the brain voxels	(i) Easy to calculate and understand	(i) Requires a priori selection of ROI, which may lead to potential biases

Regional homogeneity	Using Kendall's coefficient concordance to measure the similarity of a given voxel with its nearest neighbors based on the BOLD time-series	(i) Easy to calculate and understand	(i) Potential biases attached to prior seed selection

Amplitude of low-frequency fluctuations	Estimating the intensity of regional spontaneous brain activity by calculating the voxel-wise magnitude within a defined low-frequency range	(i) Can serve as a potential confounding variable when investigating functional connectivity and network	(i) Sensitive to physiological noise, which makes fractional ALFF (fALFF) approach a better choice

Principal component analysis	Finding spatial and temporal components that capture as much of the variability of the data based on decorrelation as possible	(i) Can verify the facticity of difference in the activations between conditions or groups without specifying any prior knowledge of the form of BOLD response or the structure of the experimental design	(i) Based on strong assumptions like linearity, orthogonal principal components, and high signal noise ratio

Independent component analysis	Separating distinct resting-state networks that are spatially or temporally independent of each other and identifying noise within the BOLD signal	(i) Can generate spatially or temporally distributed DM functional connectivity patterns with relatively few a priori assumptions	(i) May be less sensitive to interindividual variation in the composition of such networks and may be more likely to produce errors at the group level if a network is presented across multiple components in some subjects.

Graph theory	Describing the topology of the functional brain networks by calculating connectional characteristics of the graph comprised of nodes (voxels) and edges (connections between voxels)	(i) Directly describes and compares different brain networks utilizing topological parameters	(i) Difficult to interpret

## References

[1] Friston K. J., Holmes A. P., Worsley K. J., Poline J. P., Frith C. D., Frackowiak R. S. J. (1994). Statistical parametric maps in functional imaging: a general linear approach. *Human Brain Mapping*.

[2] Juckel G., Schlagenhauf F., Koslowski M. (2006). Dysfunction of ventral striatal reward prediction in schizophrenia. *NeuroImage*.

[3] Monti M. M. (2011). Statistical analysis of fMRI time-series: a critical review of the GLM approach. *Frontiers in Human Neuroscience*.

[4] Friston K. J., Buechel C., Fink G. R., Morris J., Rolls E., Dolan R. J. (1997). Psychophysiological and modulatory interactions in neuroimaging. *NeuroImage*.

[5] Das P., Kemp A. H., Flynn G. (2007). Functional disconnections in the direct and indirect amygdala pathways for fear processing in schizophrenia. *Schizophrenia Research*.

[6] Wang L., Metzak P. D., Woodward T. S. (2011). Aberrant connectivity during self-other source monitoring in schizophrenia. *Schizophrenia Research*.

[7] McIntosh A. R., Gonzalez-Lima F. (1991). Structural modeling of functional neural pathways mapped with 2-deoxyglucose: effects of acoustic startle habituation on the auditory system. *Brain Research*.

[8] Raichle M. E., MacLeod A. M., Snyder A. Z., Powers W. J., Gusnard D. A., Shulman G. L. (2001). A default mode of brain function. *Proceedings of the National Academy of Sciences of the United States of America*.

[9] Büchel C., Friston K. J. (1997). Modulation of connectivity in visual pathways by attention: cortical interactions evaluated with structural equation modelling and fMRI. *Cerebral Cortex*.

[10] Penny W. D., Stephan K. E., Mechelli A., Friston K. J. (2004). Modelling functional integration: a comparison of structural equation and dynamic causal models. *NeuroImage*.

[11] Fransson P. (2006). How default is the default mode of brain function? Further evidence from intrinsic BOLD signal fluctuations. *Neuropsychologia*.

[12] Schlösser R., Gesierich T., Kaufmann B. (2003). Altered effective connectivity during working memory performance in schizophrenia: a study with fMRI and structural equation modeling. *NeuroImage*.

[13] Friston K. J., Harrison L., Penny W. (2003). Dynamic causal modelling. *NeuroImage*.

[14] Grefkes C., Nowak D. A., Wang L. E., Dafotakis M., Eickhoff S. B., Fink G. R. (2010). Modulating cortical connectivity in stroke patients by rTMS assessed with fMRI and dynamic causal modeling. *NeuroImage*.

[15] Friston K. (2009). Causal modelling and brain connectivity in functional magnetic resonance imaging. *PLoS Biology*.

[16] Roebroeck A., Formisano E., Goebel R. (2005). Mapping directed influence over the brain using Granger causality and fMRI. *NeuroImage*.

[17] Goebel R., Roebroeck A., Kim D.-S., Formisano E. (2003). Investigating directed cortical interactions in time-resolved fMRI data using vector autoregressive modeling and Granger causality mapping. *Magnetic Resonance Imaging*.

[18] Liao W., Mantini D., Zhang Z. (2010). Evaluating the effective connectivity of resting state networks using conditional Granger causality. *Biological Cybernetics*.

[19] Granger C. W. (1988). Some recent developments in a concept of causality. *Journal of Econometrics*.

[20] Kamiński M., Ding M., Truccolo W. A., Bressler S. L. (2001). Evaluating causal relations in neural systems: Granger causality, directed transfer function and statistical assessment of significance. *Biological Cybernetics*.

[21] Demirci O., Stevens M. C., Andreasen N. C. (2009). Investigation of relationships between fMRI brain networks in the spectral domain using ICA and Granger causality reveals distinct differences between schizophrenia patients and healthy controls. *NeuroImage*.

[22] Hamilton J. P., Chen G., Thomason M. E., Schwartz M. E., Gotlib I. H. (2011). Investigating neural primacy in major depressive disorder: multivariate Granger causality analysis of resting-state fMRI time-series data. *Molecular Psychiatry*.

[23] Wang L., Shen H., Tang F., Zang Y., Hu D. (2012). Combined structural and resting-state functional MRI analysis of sexual dimorphism in the young adult human brain: an MVPA approach. *NeuroImage*.

[24] LaConte S., Strother S., Cherkassky V., Anderson J., Hu X. (2005). Support vector machines for temporal classification of block design fMRI data. *NeuroImage*.

[25] Mourão-Miranda J., Reynaud E., McGlone F., Calvert G., Brammer M. (2006). The impact of temporal compression and space selection on SVM analysis of single-subject and multi-subject fMRI data. *NeuroImage*.

[26] Serences J. T., Ester E. F., Vogel E. K., Awh E. (2009). Stimulus-specific delay activity in human primary visual cortex. *Psychological Science*.

[27] Wang Z. (2014). Support vector machine learning-based cerebral blood flow quantification for arterial spin labeling MRI. *Human Brain Mapping*.

[28] Hanson S. J., Matsuka T., Haxby J. V. (2004). Combinatorial codes in ventral temporal lobe for object recognition: Haxby (2001) revisited: is there a ‘face’ area?. *NeuroImage*.

[29] Polyn S. M., Natu V. S., Cohen J. D., Norman K. A. (2005). Category-specific cortical activity precedes retrieval during memory search. *Science*.

[30] Johnson J. D., McDuff S. G. R., Rugg M. D., Norman K. A. (2009). Recollection, familiarity, and cortical reinstatement: a multivoxel pattern analysis. *Neuron*.

[31] Carlson T. A., Schrater P., He S. (2003). Patterns of activity in the categorical representations of objects. *Journal of Cognitive Neuroscience*.

[32] Raizada R. D. S., Tsao F.-M., Liu H.-M., Holloway I. D., Ansari D., Kuhl P. K. (2010). Linking brain-wide multivoxel activation patterns to behaviour: examples from language and math. *NeuroImage*.

[33] Brodersen K. H., Wiech K., Lomakina E. I. (2012). Decoding the perception of pain from fMRI using multivariate pattern analysis. *NeuroImage*.

[34] Koster-Hale J., Saxe R., Dungan J., Young L. L. (2013). Decoding moral judgments from neural representations of intentions. *Proceedings of the National Academy of Sciences of the United States of America*.

[35] Vanhaudenhuyse A., Noirhomme Q., Tshibanda L. J.-F. (2010). Default network connectivity reflects the level of consciousness in non-communicative brain-damaged patients. *Brain*.

[36] Weil R. S., Rees G. (2010). Decoding the neural correlates of consciousness. *Current Opinion in Neurology*.

[37] Dosenbach N. U. F., Nardos B., Cohen A. L. (2010). Prediction of individual brain maturity using fMRI. *Science*.

[38] Coutanche M. N., Thompson-Schill S. L., Schultz R. T. (2011). Multi-voxel pattern analysis of fMRI data predicts clinical symptom severity. *NeuroImage*.

[39] Biswal B., Yetkin F. Z., Haughton V. M., Hyde J. S. (1995). Functional connectivity in the motor cortex of resting human brain using echo-planar MRI. *Magnetic Resonance in Medicine*.

[40] Lowe M. J., Mock B. J., Sorenson J. A. (1998). Functional connectivity in single and multislice echoplanar imaging using resting-state fluctuations. *NeuroImage*.

[41] Greicius M. D., Krasnow B., Reiss A. L., Menon V. (2003). Functional connectivity in the resting brain: a network analysis of the default mode hypothesis. *Proceedings of the National Academy of Sciences of the United States of America*.

[42] Fransson P. (2005). Spontaneous low-frequency BOLD signal fluctuations: an fMRI investigation of the resting-state default mode of brain function hypothesis. *Human Brain Mapping*.

[43] Raichle M. E., Snyder A. Z. (2007). A default mode of brain function: a brief history of an evolving idea. *NeuroImage*.

[44] Greicius M. D., Srivastava G., Reiss A. L., Menon V. (2004). Default-mode network activity distinguishes Alzheimer's disease from healthy aging: Evidence from functional MRI. *Proceedings of the National Academy of Sciences of the United States of America*.

[45] Bluhm R., Williamson P., Lanius R. (2009). Resting state default-mode network connectivity in early depression using a seed region-of-interest analysis: decreased connectivity with caudate nucleus. *Psychiatry and Clinical Neurosciences*.

[46] Zhu X., Wang X., Xiao J. (2012). Evidence of a dissociation pattern in resting-state default mode network connectivity in first-episode, treatment-naive major depression patients. *Biological Psychiatry*.

[47] Koch W., Teipel S., Mueller S. (2012). Diagnostic power of default mode network resting state fMRI in the detection of Alzheimer's disease. *Neurobiology of Aging*.

[48] Agosta F., Pievani M., Geroldi C., Copetti M., Frisoni G. B., Filippi M. (2012). Resting state fMRI in Alzheimer's disease: beyond the default mode network. *Neurobiology of Aging*.

[49] Uddin L. Q., Kelly A. M. C., Biswal B. B. (2008). Network homogeneity reveals decreased integrity of default-mode network in ADHD. *Journal of Neuroscience Methods*.

[50] Assaf M., Jagannathan K., Calhoun V. D. (2010). Abnormal functional connectivity of default mode sub-networks in autism spectrum disorder patients. *NeuroImage*.

[51] Greicius M. D., Flores B. H., Menon V. (2007). Resting-state functional connectivity in major depression: abnormally increased contributions from subgenual cingulate cortex and thalamus. *Biological Psychiatry*.

[52] Song M., Zhou Y., Li J. (2008). Brain spontaneous functional connectivity and intelligence. *NeuroImage*.

[53] Larson-Prior L. J., Zempel J. M., Nolan T. S., Prior F. W., Snyder A., Raichle M. E. (2009). Cortical network functional connectivity in the descent to sleep. *Proceedings of the National Academy of Sciences of the United States of America*.

[54] Andrews-Hanna J. R., Snyder A. Z., Vincent J. L. (2007). Disruption of large-scale brain systems in advanced aging. *Neuron*.

[55] Ranganath C., Heller A., Cohen M. X., Brozinsky C. J., Rissman J. (2005). Functional connectivity with the hippocampus during successful memory formation. *Hippocampus*.

[56] Whitfield-Gabrieli S., Thermenos H. W., Milanovic S. (2009). Hyperactivity and hyperconnectivity of the default network in schizophrenia and in first-degree relatives of persons with schizophrenia. *Proceedings of the National Academy of Sciences of the United States of America*.

[57] Fox M. D., Snyder A. Z., Vincent J. L., Corbetta M., van Essen D. C., Raichle M. E. (2005). The human brain is intrinsically organized into dynamic, anticorrelated functional networks. *Proceedings of the National Academy of Sciences of the United States of America*.

[58] Zhou Y., Liang M., Tian L. (2007). Functional disintegration in paranoid schizophrenia using resting-state fMRI. *Schizophrenia Research*.

[59] Yu R., Liu B., Wang L., Chen J., Liu X. (2013). Enhanced functional connectivity between putamen and supplementary motor area in Parkinson's disease patients. *PLoS ONE*.

[60] Yu R., Gollub R. L., Spaeth R., Napadow V., Wasan A., Kong J. (2014). Disrupted functional connectivity of the periaqueductal gray in chronic low back pain. *NeuroImage: Clinical*.

[61] Cole D. M., Smith S. M., Beckmann C. F. (2010). Advances and pitfalls in the analysis and interpretation of resting-state FMRI data. *Frontiers in Systems Neuroscience*.

[62] Zang Y., Jiang T., Lu Y., He Y., Tian L. (2004). Regional homogeneity approach to fMRI data analysis. *NeuroImage*.

[63] Yu R., Zhao L., Tian J. (2013). Regional homogeneity changes in heavy male smokers: a resting-state functional magnetic resonance imaging study. *Addiction Biology*.

[64] Zhang Z., Liu Y., Jiang T. (2012). Altered spontaneous activity in Alzheimer's disease and mild cognitive impairment revealed by Regional Homogeneity. *NeuroImage*.

[65] Yao Z., Wang L., Lu Q., Liu H., Teng G. (2009). Regional homogeneity in depression and its relationship with separate depressive symptom clusters: a resting-state fMRI study. *Journal of Affective Disorders*.

[66] Liu Z., Xu C., Xu Y. (2010). Decreased regional homogeneity in insula and cerebellum: a resting-state fMRI study in patients with major depression and subjects at high risk for major depression. *Psychiatry Research: Neuroimaging*.

[67] Liu H., Liu Z., Liang M. (2006). Decreased regional homogeneity in schizophrenia: a resting state functional magnetic resonance imaging study. *NeuroReport*.

[68] Wu T., Long X., Zang Y. (2009). Regional homogeneity changes in patients with parkinson's disease. *Human Brain Mapping*.

[69] Shukla D. K., Keehn B., Müller R. A. (2010). Regional homogeneity of fMRI time series in autism spectrum disorders. *Neuroscience Letters*.

[70] Paakki J.-J., Rahko J., Long X. (2010). Alterations in regional homogeneity of resting-state brain activity in autism spectrum disorders. *Brain Research*.

[71] Wang L., Song M., Jiang T., Zhang Y., Yu C. (2011). Regional homogeneity of the resting-state brain activity correlates with individual intelligence. *Neuroscience Letters*.

[72] Liu C., Liu Y., Li W. (2011). Increased regional homogeneity of blood oxygen level-dependent signals in occipital cortex of early blind individuals. *NeuroReport*.

[73] Liu J., Gao X.-P., Osunde I. (2010). Increased regional homogeneity in internet addiction disorder: a resting state functional magnetic resonance imaging study. *Chinese Medical Journal*.

[74] Yu R., Hsieh M. H., Wang H. L. S. (2013). Frequency dependent alterations in regional homogeneity of baseline brain activity in schizophrenia. *PLoS ONE*.

[75] Guo S., Kendrick K. M., Yu R., Wang H.-L. S., Feng J. (2014). Key functional circuitry altered in schizophrenia involves parietal regions associated with sense of self. *Human Brain Mapping*.

[76] Guo S., Kendrick K. M., Zhang J. (2013). Brain-wide functional inter-hemispheric disconnection is a potential biomarker for schizophrenia and distinguishes it from depression. *NeuroImage: Clinical*.

[77] Zou Q.-H., Zhu C.-Z., Yang Y. (2008). An improved approach to detection of amplitude of low-frequency fluctuation (ALFF) for resting-state fMRI: fractional ALFF. *Journal of Neuroscience Methods*.

[78] Zuo X.-N., Di Martino A., Kelly C. (2010). The oscillating brain: complex and reliable. *NeuroImage*.

[79] Yang H., Long X.-Y., Yang Y. (2007). Amplitude of low frequency fluctuation within visual areas revealed by resting-state functional MRI. *NeuroImage*.

[80] Liu J., Ren L., Womer F. Y. (2014). Alterations in amplitude of low frequency fluctuation in treatment-naïve major depressive disorder measured with resting-state fMRI. *Human Brain Mapping*.

[81] Zang Y.-F., He Y., Zhu C.-Z. (2007). Altered baseline brain activity in children with ADHD revealed by resting-state functional MRI. *Brain & Development*.

[82] Bing X., Qiu M.-G., Ye Z. (2013). Alterations in the cortical thickness and the amplitude of low-frequency fluctuation in patients with post-traumatic stress disorder. *Brain Research*.

[83] Hu S., Chao H. H.-A., Zhang S., Ide J. S., Li C.-S. R. (2014). Changes in cerebral morphometry and amplitude of low-frequency fluctuations of BOLD signals during healthy aging: correlation with inhibitory control. *Brain Structure and Function*.

[84] Hoptman M. J., Zuo X.-N., Butler P. D. (2010). Amplitude of low-frequency oscillations in schizophrenia: a resting state fMRI study. *Schizophrenia Research*.

[85] Yu R., Chien Y.-L., Wang H.-L. S. (2014). Frequency-specific alternations in the amplitude of low-frequency fluctuations in schizophrenia. *Human Brain Mapping*.

[86] Zuo X. N., di Martino A., Kelly C. (2010). The oscillating brain: complex and reliable. *NeuroImage*.

[87] Friston K. J., Frith C. D., Liddle P. F., Frackowiak R. S. J. (1993). Functional connectivity: the principal-component analysis of large (PET) data sets. *Journal of Cerebral Blood Flow and Metabolism*.

[88] Pearson K. (1901). LIII. On lines and planes of closest fit to systems of points in space. *Philosophical Magazine Series 6*.

[89] Wold S., Esbensen K., Geladi P. (1987). Principal component analysis. *Chemometrics and Intelligent Laboratory Systems*.

[90] Viviani R., Grön G., Spitzer M. (2005). Functional principal component analysis of fMRI data. *Human Brain Mapping*.

[91] Shen H., Wang L., Liu Y., Hu D. (2010). Discriminative analysis of resting-state functional connectivity patterns of schizophrenia using low dimensional embedding of fMRI. *NeuroImage*.

[92] Shlens J. A tutorial on principal component analysis. http://arxiv.org/abs/1404.1100.

[93] McKeown M. J., Sejnowski T. J. (1998). Independent component analysis of fMRI data: examining the assumptions. *Human Brain Mapping*.

[94] Calhoun V. D., Adali T., Pearlson G. D., Pekar J. J. (2001). A method for making group inferences from functional MRI data using independent component analysis. *Human Brain Mapping*.

[95] Smith S. M., Fox P. T., Miller K. L. (2009). Correspondence of the brain's functional architecture during activation and rest. *Proceedings of the National Academy of Sciences of the United States of America*.

[96] Beckmann C. F., DeLuca M., Devlin J. T., Smith S. M. (2005). Investigations into resting-state connectivity using independent component analysis. *Philosophical Transactions of the Royal Society B: Biological Sciences*.

[97] Hyvärinen A., Karhunen J., Oja E. (2004). *Independent Component Analysis*.

[98] Bannistert P. R., Beckmann C. F., Jenkinson M., Smith S., Brady J. M. Motion artefact decorrelation in fMRI analysis using ICA.

[99] Perlbarg V., Bellec P., Anton J.-L., Pélégrini-Issac M., Doyon J., Benali H. (2007). CORSICA: correction of structured noise in fMRI by automatic identification of ICA components. *Magnetic Resonance Imaging*.

[100] van de Ven V. G., Formisano E., Prvulovic D., Roeder C. H., Linden D. E. J. (2004). Functional connectivity as revealed by spatial independent component analysis of fMRI measurements during rest. *Human Brain Mapping*.

[101] Damoiseaux J. S., Rombouts S. A. R. B., Barkhof F. (2006). Consistent resting-state networks across healthy subjects. *Proceedings of the National Academy of Sciences of the United States of America*.

[102] Schöpf V., Windischberger C., Robinson S. (2011). Model-free fMRI group analysis using FENICA. *NeuroImage*.

[103] Hampson M., Peterson B. S., Skudlarski P., Gatenby J. C., Gore J. C. (2002). Detection of functional connectivity using temporal correlations in MR images. *Human Brain Mapping*.

[104] Sporns O. (2003). Graph theory methods for the analysis of neural connectivity patterns. *Neuroscience Databases*.

[105] Bullmore E., Sporns O. (2009). Complex brain networks: graph theoretical analysis of structural and functional systems. *Nature Reviews Neuroscience*.

[106] van den Heuvel M. P., Hulshoff Pol H. E. (2010). Exploring the brain network: a review on resting-state fMRI functional connectivity. *European Neuropsychopharmacology*.

[107] van den Heuvel M. P., Stam C. J., Boersma M., Hulshoff Pol H. E. (2008). Small-world and scale-free organization of voxel-based resting-state functional connectivity in the human brain. *NeuroImage*.

[108] Bassett D. S., Bullmore E. (2006). Small-world brain networks. *The Neuroscientist*.

[109] Wang J., Zuo X., He Y. (2010). Graph-based network analysis of resting-state functional MRI. *Frontiers in Systems Neuroscience*.

[110] Bernhardt B. C., Chen Z., He Y., Evans A. C., Bernasconi N. (2011). Graph-theoretical analysis reveals disrupted small-world organization of cortical thickness correlation networks in temporal lobe epilepsy. *Cerebral Cortex*.

[111] He Y., Chen Z. J., Evans A. C. (2007). Small-world anatomical networks in the human brain revealed by cortical thickness from MRI. *Cerebral Cortex*.

[112] Iturria-Medina Y., Canales-Rodríguez E. J., Melie-García L. (2007). Characterizing brain anatomical connections using diffusion weighted MRI and graph theory. *NeuroImage*.

[113] Iturria-Medina Y., Sotero R. C., Canales-Rodríguez E. J., Alemán-Gómez Y., Melie-García L. (2008). Studying the human brain anatomical network via diffusion-weighted MRI and Graph Theory. *NeuroImage*.

[114] Meunier D., Achard S., Morcom A., Bullmore E. (2009). Age-related changes in modular organization of human brain functional networks. *NeuroImage*.

[115] Gong G., Rosa-Neto P., Carbonell F., Chen Z. J., He Y., Evans A. C. (2009). Age- and gender-related differences in the cortical anatomical network. *The Journal of Neuroscience*.

[116] He Y., Dagher A., Chen Z. (2009). Impaired small-world efficiency in structural cortical networks in multiple sclerosis associated with white matter lesion load. *Brain*.

[117] Shu N., Liu Y., Li K. (2011). Diffusion tensor tractography reveals disrupted topological efficiency in white matter structural networks in multiple sclerosis. *Cerebral Cortex*.

[118] Buckner R. L., Sepulcre J., Talukdar T. (2009). Cortical hubs revealed by intrinsic functional connectivity: mapping, assessment of stability, and relation to Alzheimer's disease. *Journal of Neuroscience*.

[119] Sanz-Arigita E. J., Schoonheim M. M., Damoiseaux J. S. (2010). Loss of ‘small-world’ networks in Alzheimer's disease: graph analysis of FMRI resting-state functional connectivity. *PLoS ONE*.

[120] Yao Z., Zhang Y., Lin L. (2010). Abnormal cortical networks in mild cognitive impairment and Alzheimer's disease. *PLoS Computational Biology*.

[121] van den Heuvel M. P., Mandl R. C. W., Stam C. J., Kahn R. S., Hulshoff Pol H. E. (2010). Aberrant frontal and temporal complex network structure in schizophrenia: a graph theoretical analysis. *The Journal of Neuroscience*.

[122] Micheloyannis S., Pachou E., Stam C. J. (2006). Small-world networks and disturbed functional connectivity in schizophrenia. *Schizophrenia Research*.

[123] Bassett D. S., Bullmore E., Verchinski B. A., Mattay V. S., Weinberger D. R., Meyer-Lindenberg A. (2008). Hierarchical organization of human cortical networks in health and Schizophrenia. *The Journal of Neuroscience*.

[124] Ajilore O., Lamar M., Leow A., Zhang A., Yang S., Kumar A. (2014). Graph theory analysis of cortical-subcortical networks in late-life depression. *The American Journal of Geriatric Psychiatry*.

[125] Lim H. K., Jung W. S., Aizenstein H. J. (2013). Aberrant topographical organization in gray matter structural network in late life depression: a graph theoretical analysis. *International Psychogeriatrics*.

[126] Ponten S. C., Bartolomei F., Stam C. J. (2007). Small-world networks and epilepsy: graph theoretical analysis of intracerebrally recorded mesial temporal lobe seizures. *Clinical Neurophysiology*.

